# TEG® and RapidTEG® are unreliable for detecting warfarin-coagulopathy: a prospective cohort study

**DOI:** 10.1186/1477-9560-12-4

**Published:** 2014-02-04

**Authors:** C Michael Dunham, Charlene Rabel, Barbara M Hileman, Jason Schiraldi, Elisha A Chance, Mark T Shima, Alddo A Molinar, David A Hoffman

**Affiliations:** 1 Trauma/Critical Services, St. Elizabeth Health Center, 1044 Belmont Avenue, Youngstown, OH 44501, USA; 2 Central Laboratory Services, St. Elizabeth Health Center, 1044 Belmont Avenue, Youngstown, OH 44501, USA; 3 Electrophysiology Service, St. Elizabeth Health Center, 1044 Belmont Avenue, Youngstown, OH 44501, USA; 4 Department of Anesthesiology, St. Elizabeth Health Center, 1044 Belmont Avenue, Youngstown, OH 44501, USA; 5 Division of Cardiology, St. Elizabeth Health Center, 1044 Belmont Avenue, Youngstown, OH 44501, USA

**Keywords:** Warfarin, Thromboelastography, Coagulopathy

## Abstract

**Background:**

Thromboelastography® (TEG) utilizes kaolin, an intrinsic pathway activator, to assess clotting function. Recent published studies suggest that TEG results are commonly normal in patients receiving warfarin, despite an increased International Normalized Ratio (INR). Because RapidTEG™ includes tissue factor, an extrinsic pathway activator, as well as kaolin, we hypothesized that RapidTEG would be more sensitive in detecting a warfarin-effect.

**Methods:**

Included in this prospective study were 22 consecutive patients undergoing elective cardioversion and receiving warfarin. Prior to cardioversion, blood was collected to assess INR, Prothrombin Time, TEG, and RapidTEG.

**Results:**

INR Results: 2.8 ± 0.5 (1.6 to 4.2). Prothrombin Time Results: 19.1 ± 2.2 (13.9. to 24.3).

TEG Results (Reference Range): **R-Time**: 8.3 ± 2.7 (2–8); **K-Time**: 2.1 ± 1.4 (1–3); **Angle**: 62.5 ± 10.3 (55–78); **MA**: 63.2 ± 10.3 (51–69); **G**: 9.4 ± 3.5 (4.6-10.9); R-Time within normal range: 10 (45.5%) with INR 2.9 ± 0.3; Correlation coefficients for INR and each of the 5 TEG variables were insignificant (*P* > 0.05).

RapidTEG Results (Reference Range): **ACT**: 132 ± 58 (86–118); **K-Time**: 1.2 ± 0.5 (1–2); **Angle**: 75.4 ± 5.2 (64–80); **MA**: 63.4 ± 5.1 (52–71); **G**: 8.9 ± 2.0 (5.0-11.6); ACT within normal range: 9 (40.9%) with INR 2.7 ± 0.5; Correlation coefficients for INR and each of the 5 RapidTEG variables were insignificant (*P* > 0.05).

**Conclusions:**

TEG, using kaolin activation, and RapidTEG, with kaolin and tissue factor activation, were normal in a substantial percent of warfarin patients, despite an increased INR. The false-negative rate for detecting warfarin coagulopathy with either test is unacceptable. The lack of correlation between INR and all TEG and RapidTEG components further indicates that these methodologies are insensitive to warfarin effects. Findings suggest that intrinsic pathway activation may mitigate detection of an extrinsic pathway coagulopathy.

## Background

The prevalence of warfarin use in the United States is unknown, however the Food and Drug Administration estimates that more than 31 million prescriptions for warfarin were written in 2004 [1]. A publication by Dossett indicates that warfarin use is common among injured patients and its prevalence has increased each year since 2002 [2]. In that study, warfarin was found to be associated with a significant increase in trauma-related mortality, even after adjusting for confounding co-morbidities.

Thromboelastography (TEG®) assesses the viscoelastic properties of blood during the clotting process [3-7]. The R-Time (clotting time) is the time in minutes from clot activation until the graphic amplitude is 2 mm, that is, until the first detectable levels of fibrin clot formation [3,4,6,7]. It is important to recognize that with the TEG assay, kaolin or celite, as factor XII activators, or tissue factor may be used to enhance clot formation [3,7]. Several review articles emphasized the importance of knowing the “assay variant” (clotting activator) utilized in a given set of studies [4-6]. In other words, several different reagents can be used: Kaolin – Intrinsic Activator; Tissue Factor – Extrinsic Activator; and others [5,6]. Thus, depending on the activator, this may correspond to clotting times measured by aPTT (intrinsic process) or PT (extrinsic process)” [4]. More specifically, kaolin is an intrinsic activator; therefore coagulation time (R-Time) is sensitive to heparin [4].

Recent literature suggests that viscoelastic study results vary, when there is a warfarin-effect, depending on the activator being used. Of relevance, a study by Rumph demonstrated that Thromboelastometry (ROTEM®), using calcium chloride and tissue factor activation, showed marked increases in Clotting Time (analogous to TEG R-Time), when warfarin-treated plasma was analyzed [8]. Warfarin Clotting Time was 289 ± 78 seconds in the warfarin-treated plasma, with a normal value of 38–79 seconds. In contradistinction, a recently published study demonstrated that kaolin-activated TEG results were typically normal in patients on warfarin and with a therapeutic International Normalized Ratio (INR) [9]. Of eight patients taking warfarin and with therapeutic INR values, only one patient had an abnormal TEG.

In an isolated case at our institution, a patient on warfarin prior to hospital admission was admitted to the surgical intensive care unit and found to have INR values of five and 11 within 60 minutes of each other. The patient had a large retroperitoneal hematoma by CT scan, severe hypotension, and profound lactic acidosis. Two TEG Reaction (R)-Time values were within the normal range at the same times that the INR values were severely elevated. This compellingly suggested to the first author that TEG, with kaolin activation, may be insensitive to warfarin-effect. To investigate the sensitivity of kaolin-activated TEG to warfarin-effect, we evaluated the Prothrombin Time (PT), INR, and TEG values in patients on warfarin who were undergoing elective cardioversion. Because the RapidTEG™ (rTEG) assay includes tissue factor activation, in addition to kaolin, rTEG was also performed. We hypothesized that TEG would be relatively insensitive to warfarin-coagulopathy, whereas rTEG would have improved and acceptable sensitivity.

## Methods

This is a study of patients receiving Wafarin therapy who were scheduled for elective cardioversion with prospective laboratory evaluation of INR, PT, and standard parameters of both TEG (kaolin activation) and rTEG (kaolin and tissue factor activation). TEG parameters assessed were R-Time, Kinetics (K)-Time, alpha-angle, maximum amplitude (MA), and G-value. rTEG parameters evaluated included Activated Clotting Time (ACT [R-Time derivative]), K-Time, alpha-angle, MA, and G-value. When INR was > 1.5 and TEG R-Time was > 8.0, the established upper limit of normal, TEG was considered to have a true-positive response. When INR was > 1.5 and TEG R-Time was ≤ 8.0, TEG was considered to have a false-negative response. When INR was > 1.5 and ACT was > 118, the established upper limit of normal, rTEG was considered to have a true-positive response. When INR was > 1.5 and ACT was ≤ 118, rTEG was considered to have a false-negative response.

The St. Elizabeth Health Center Institutional Review Board approved this study, with consent waiver, and included patients who presented for elective cardioversion at St. Elizabeth’s Health Center between July 27, 2012 and October 9, 2012. Patients included in the study were those greater than 18 years of age, who were receiving continuous Wafarin therapy. Patients excluded from the study were those who were taking other anticoagulant medications or platelet inhibitors such as Aspirin, Ticlopidine, Clopidogrel, Abciximab, Tirofiban, and Eptifibatide, un-fractionated or low-molecular-weight heparin, Dabigatran, and Argatroban.

A 4.5 mL sample of whole blood was collected into a buffered sodium citrate phlebotomy vial at the point of care one-hour prior to cardioversion. The blood was then transported to a College of American Pathologists and Clinical Laboratory Improvement Amendments certified laboratory, where the samples were processed and analyzed. Using manufacturer specified techniques, TEG and rTEG analyses were performed on a Thromboelastograph Whole Blood Coagulation Analyzer (Model 5000, Haemoscope Corporation, Niles, Il, USA). Each specimen was maintained at room temperature for 15 minutes after collection to equilibrate. Normal and abnormal controls were run for TEG/rTEG and PT/INR per manufacturer guidelines prior to testing patient samples. Approximately 2 mL of citrated whole blood was run on the TEG/rTEG analyzer per manufacturer instructions by an American Society for Clinical Pathology certified Medical Technologist. The remaining 2.5 mL of citrated whole blood was centrifuged at 3,500 rpm for 10 minutes. The plasma was then analyzed for PT and INR on an ACL TOP® 500 Hemostasis Testing System (Instrumentation Laboratories, Belgium) per manufacturer instructions by a certified Medical Technologist. TEG/rTEG and PT/INR were performed from the same citrated sample within one hour of collection. No specimen was hemolyzed.

The results were entered into Microsoft Excel and imported into SAS System for Windows, release 9.2 (SAS Institute Inc., Cary, NC, USA) to perform the statistical analysis. *P* value of < 0.05 represented statistical significance. Correlation coefficient analyses were performed between the INR and each of the TEG and rTEG parameter values. The sensitivity for detecting an elevated INR was computed for each TEG and rTEG parameter. Specifically, a rate was determined for each TEG and rTEG parameter to determine if the values were above the upper limit of the normal range, as reported by the device manufacturer from healthy volunteers. TEG and rTEG analysis kits were donated by the Haemoscope Corporation.

## Results

During July 2012 through October, 2012, a total of 22 consecutive patients undergoing elective cardioversion had an increased INR, due to warfarin. For the 22 patients, INR was 2.8 ± 0.5 (1.6 to 4.2) and PT was 19.1 ± 2.2 (13.9. to 24.3). TEG results with reference ranges are in Table 1. TEG R-Time value was above the upper reference range of 8.0 (true-positive) in 12/22, indicating that TEG sensitivity for warfarin coagulopathy was 54.5% (95% CI: 34.5-73.1%). Accordingly, the false-negative rate (10/22) for warfarin coagulopathy (INR 2.9 ± 0.3) was 45.5% (95% CI: 25.8-65.5%). *P*-values for INR and TEG correlations were as follows: R-Time *P* = 0.7657, K-Time *P* = 0.8336, alpha-angle *P* = 0.9783, MA *P* = 0.7057, G-value *P* = 0.9818.

**Table 1:** INR, TEG and rTEG results and reference ranges

**Parameter**	**Mean ± SD**	**Reference range**
**INR**	2.8 ± 0.5	< 1.5
**TEG R-Time**	8.3 ± 2.7	2-8
**TEG K-Time**	2.1 ± 1.4	1-3.0
**TEG alpha-angle**	62.5 ± 10.3	55-78
**TEG MA**	63.2 ± 10.3	51-69
**TEG G-value**	9.4 ± 3.5	4.6-10.9
**rTEG ACT**	132 ± 57.7	86-118
**rTEG K-Time**	1.2 ± 0.5	1-2.0
**rTEG alpha-angle**	75.4 ± 5.2	64-80.0
**rTEG MA**	63.4 ± 5.1	52-71.0
**rTEG G-value**	8.9 ± 2.0	5.0-11.6

INR, International Normalized Ration; TEG, Thromboelastography; rTEG, RapidTEG; R-Time, Reaction Time; K-Time, Kinetics Time; MA, maximum amplitude; G-value, clot strength/elasticity; ACT, activated clotting time.

RapidTEG results with reference ranges are in Table 1. RapidTEG ACT-value was above the upper reference range of 118 (true-positive) in 13/22, suggesting that RapidTEG sensitivity for warfarin coagulopathy was 59.1% (95% CI: 38.5-76.8%). Apropos, the false-negative rate (9/22) for warfarin coagulopathy (INR 2.7 ± 0.5) was 40.9% (95% CI: 23.2-61.5%). *P*-values for INR and RapidTEG correlations were as follows: ACT *P* = 0.7700, K-Time *P* = 0.4710, alpha-angle *P* = 0.1167, MA *P* = 0.5682, G-value *P* = 0.4554.

## Discussion

Although patients in the current study had a substantial warfarin-effect according to PT and INR values, the kaolin-activated TEG R-Time values were within the normal range in nearly half. The other TEG parameters (K-Time, alpha-angle, MA, and G-value) were virtually always within the normal range. Additionally, there were no significant correlations between any of the five TEG variables and the INR values. Contrary to our hypothesis, the kaolin plus tissue factor activated rTEG results revealed similar findings. Thus, we believe that TEG and rTEG sensitivity for detecting a warfarin-effect is clinically unacceptable. Because kaolin is common to TEG and rTEG, it appears that intrinsic system activation is suboptimal for detecting alterations in the extrinsic system warfarin produces.

Circumstances exist, in institutions with TEG-availability, where simultaneous INR and TEG or RapidTEG testing may occur. Alternatively, a TEG may be obtained, but an INR test has not been recently performed. A clinician might be influenced by the TEG result of a patient receiving warfarin, if they believe that TEG provides an accurate appraisal of warfarin-effect. In either circumstance, the clinician needs to be aware of the relative insensitivity of TEG and RapidTEG for assessing warfarin-coagulopathy.

### Literature documenting insensitivity of TEG to warfarin

In addition to the current study, other investigators specifically indicate that TEG is insensitive to warfarin-effects. The Nascimento study found that only one of eight therapeutic warfarin-patients had an abnormal TEG [9]. In a prospective study of patients given warfarin, the mean TEG R-Time remained in the normal range [10]. Although the number of TEG publications has nearly tripled in the past five years, when compared to the previous five years, there is a dearth of information regard its use for detecting warfarin-effects. Apropos, there is no mention of warfarin or Coumadin in five of six recent TEG-ROTEM review articles [3-7]. However, Reikvam, in a recent TEG review article, states “The method is not sensitive to Factor VII deficiency and is not suitable for monitoring vitamin K antagonist treatment” [11]. These findings appear to represent a major knowledge deficiency in the understanding of viscoelastic hemostatic technologies for detecting warfarin.

#### ***Nuances and intricacies of TEG***

The literature includes five recent review articles that describe nuances and intricacies regarding TEG methodology, benefits, and limitations [3-7]. The reviews describe a plethora of testing details and caveats necessary for proper testing and interpretation. A couple of the TEG review articles have content that may leave the superficial reader with false impressions. One articles states “The advantage of these techniques is that they have the potential to measure the entire clotting process, …….” [3]. A statement in another review also appears to be an over generalization: “The trace is also influenced by pharmacological agents such as anticoagulants, antiplatelet therapy, and coagulation factor supplementation” [6]. Although both manuscripts subsequently provide appropriate caveats, a cursory perusal may leave the reader with a limited picture of process details and an improper interpretation of the results. In their reviews, Lang [4] and Chen [7] indicate that TEG cannot provide a comprehensive appraisal for all aspects of hemostatic function.

#### ***Conventional coagulation tests and viscoelastic hemostatic analysis***

Conventional coagulation tests (PT and activated partial thromboplastin time) at best determine only the thrombin-generation phase [4]. Further, these conventional coagulation parameters are primarily intended to detect substances acting on coagulation (vitamin K antagonists and un-fractionated heparin)” [4]. In a recent clinical study of trauma-induced coagulopathy using ROTEM, the correlations between coagulation time in EXTEM/INTEM and PT/aPTT were rather poor (r = 0.47-0.53) [5]. As well, correlations for TEG R-Times with aPTT and PT have been described as weak [5].

#### ***Study limitations***

A larger number of tested patients might have produced slightly different sensitivity and false-negative rates, with attendant 95% confidence intervals. The absence of patients with normal hemostatic function prohibited the computation of TEG and rTEG specificity and accuracy for warfarin-effect. Correlation coefficients between INR and the TEG and rTEG parameter values may have been different had persons with normal hemostatic function been included. The performance of tissue factor-TEG assays, without kaolin, would have been elucidating.

## Conclusions

TEG, using kaolin activation, and rTEG, with kaolin and tissue factor activation, were normal in a substantial percent of warfarin patients, despite an increased INR. The false-negative rate for detecting warfarin coagulopathy with either test is clinically unacceptable. The lack of correlation between INR and all TEG and rTEG components further indicates that these methodologies are insensitive to warfarin-effects. The study findings suggest that intrinsic pathway activation may mitigate detection of an extrinsic pathway coagulopathy. Current investigation results will help to fill an apparent literature void regarding warfarin-assessment during viscoelastic hemostatic assessment. The investigation results are relevant for the clinicians, hematologists, and pathologists, when considering that TEG is commonly used by the international medical community and that there are literally millions of patients receiving warfarin.

## Abbreviations

ACT: Activated clotting time; G-value: Clot strength/elasticity; INR: International Normalized Ratio; K-Time: Kinetics Time; MA: Maximum amplitude; PT: Prothrombin time; rTEG: RapidTEG™; R-Time: Reaction-Time; ROTEM®: Thromboelastometry; TEG®: Thromboelastography.

## Competing interests

TEG and rTEG assays kits were supplied by the Haemoscope Corporation. The Haemoscope Corporation had no influence on data interpretation or manuscript development. There are no financial or non-financial competing interests.

## Authors’ contributions

CMD, CR, BMH, EC, AAM, JS, and DAH conceptualized and designed the study. CMD, CR, BMH, EC, and JS were involved in the day-to-day oversight of the study. CR, BMH, EC, and JS performed the data collection. CMD performed the data analysis. CMD, CR, BMH, JS, EC, MTS, AAM, and DAH performed the data interpretation. CMD, BMH, and MTS performed the literature search and drafted the manuscript. CMD, CR, BMH, JS, EC, MTS, AAM, and DAH critically revised the manuscript for important intellectual content. All authors made substantial contributions to conception and design, or acquisition of data, or analysis and interpretation of data. All authors have been involved in drafting the manuscript or revising it critically for important intellectual content. All authors read and approved the final manuscript.
